# The Metabolic Landscape of Thymic T Cell Development *In Vivo* and *In Vitro*

**DOI:** 10.3389/fimmu.2021.716661

**Published:** 2021-07-28

**Authors:** Victoria Sun, Mark Sharpley, Karolina E. Kaczor-Urbanowicz, Patrick Chang, Amélie Montel-Hagen, Shawn Lopez, Alexandre Zampieri, Yuhua Zhu, Stéphanie C. de Barros, Chintan Parekh, David Casero, Utpal Banerjee, Gay M. Crooks

**Affiliations:** ^1^Department of Pathology & Laboratory Medicine, David Geffen School of Medicine, University of California Los Angeles (UCLA), Los Angeles, CA, United States; ^2^Molecular Biology Interdepartmental Program, UCLA, Los Angeles, CA, United States; ^3^Department of Molecular, Cell and Developmental Biology, UCLA, Los Angeles, CA, United States; ^4^Division of Oral Biology & Medicine, School of Dentistry, UCLA, Los Angeles, CA, United States; ^5^Institute for Quantitative and Computational Biosciences, UCLA, Los Angeles, CA, United States; ^6^Cancer and Blood Disease Institute, Children’s Hospital Los Angeles, Los Angeles, CA, United States; ^7^Department of Pediatrics, Keck School of Medicine, University of Southern California, Los Angeles, CA, United States; ^8^F. Widjaja Foundation Inflammatory Bowel and Immunobiology Research Institute, Cedars- Sinai Medical Center, Los Angeles, CA, United States; ^9^Department of Biological Chemistry, UCLA, Los Angeles, CA, United States; ^10^Eli and Edythe Center of Regenerative Medicine and Stem Cell Research, UCLA, Los Angeles, CA, United States; ^11^Jonsson Comprehensive Cancer Center, UCLA, Los Angeles, CA, United States; ^12^Division of Pediatric Hematology-Oncology, Department of Pediatrics, David Geffen School of Medicine, UCLA, Los Angeles, CA, United States

**Keywords:** thymus, metabolism, T cell, organoid, thymopoiesis, human, mouse, *in vitro*

## Abstract

Although metabolic pathways have been shown to control differentiation and activation in peripheral T cells, metabolic studies on thymic T cell development are still lacking, especially in human tissue. In this study, we use transcriptomics and extracellular flux analyses to investigate the metabolic profiles of primary thymic and *in vitro*-derived mouse and human thymocytes. Core metabolic pathways, specifically glycolysis and oxidative phosphorylation, undergo dramatic changes between the double-negative (DN), double-positive (DP), and mature single-positive (SP) stages in murine and human thymus. Remarkably, despite the absence of the complex multicellular thymic microenvironment, *in vitro* murine and human T cell development recapitulated the coordinated decrease in glycolytic and oxidative phosphorylation activity between the DN and DP stages seen in primary thymus. Moreover, by inducing *in vitro* T cell differentiation from *Rag1^-/-^* mouse bone marrow, we show that reduced metabolic activity at the DP stage is independent of TCR rearrangement. Thus, our findings suggest that highly conserved metabolic transitions are critical for thymic T cell development.

## Introduction

There is a rapidly growing body of evidence that metabolic changes have a key role in regulating the function, differentiation, and T cell receptor (TCR)-mediated activation of mature peripheral T cells (1–7). However, far less is known about the role of metabolism during thymopoiesis, where profound shifts in transcription, cues from the thymic microenvironment, and TCR rearrangement and signaling all combine to direct the development of a diverse array of T cells from uncommitted progenitors (8–11). Metabolic studies of human thymopoiesis are particularly lacking due to the technical challenges of using primary human tissue for functional assays.

After seeding the thymus organ, hematopoietic stem and progenitor cells (HSPCs) progressively commit to a T cell fate. The earliest thymocyte stages are called “double negative” (DN), reflecting the absence of expression for both CD4 and CD8 surface markers. Murine thymocytes progressively committing to the T cell lineage are characterized as DN1, DN2, DN3, DN4; subsequent acquisition of CD8 expression leads to the immature single positive CD8 (ISP8) stage. In human thymus, early DN progenitors express CD34 and can be further defined as Thy1, Thy2, and Thy3 (12). In both mouse and human, DN1/Thy1 populations are the earliest thymic progenitors and have multilineage potential similar to that of bone marrow multipotent progenitors (12, 13). In contrast to murine thymopoiesis, the stage that follows the early Thy1-3 progenitors in human thymopoiesis is marked by acquisition of CD4 (ISP4) (14–16). Committed thymocytes successfully rearrange the T cell receptor beta subunit (TCRβ) by the DN3 stage in mouse thymus and the ISP4 stage in human thymus (15, 17, 18). After the ISP stage, thymocytes co-express both CD4 and CD8, becoming so-called “double positive” (DP). After DP cells proceed through positive selection, they generate single-positive CD8 (SP8) or CD4 (SP4) T cells.

Current understanding of thymocyte metabolism has been mostly shaped by transcriptional and genetic perturbation studies in murine thymus. The Immunological Genome Project Consortium (ImmGen) has described a global transcriptional shutdown of metabolic genes at the DP stage in murine thymocytes that was not a result of exit from the cell cycle, but instead associated with downregulation of ribosomal proteins, RNA translation, and *Myc* expression (13). Independent studies have shown that perturbation of genes involved in metabolic pathways, such as *Glut1*, *Raptor*, *Pkm2*, *Pparδ*, *Mpc1*, *Opa1*, and *Rpl22*, disrupt the DN to DP transition in murine thymocytes (19–25). Metabolic transitions appear to be critical for proper T cell development yet have not been investigated in-depth in human thymopoiesis. In addition, most studies have primarily focused on the metabolism of either early thymocyte stages or mature T cells in the thymus separately (26); a complete characterization of metabolic shifts from the DN-SP stages has not yet been performed in mouse or human primary thymus.

The thymopoiesis field has greatly benefited from *in vitro* models of T cell differentiation from HSPCs. One such model is the Artificial Thymic Organoid (ATO), a serum-free system that generates mature T cells from various HSPC sources, such as mouse bone marrow (27), human cord blood (28), and induced human pluripotent stem cells (iPSCs) (29). Considering the challenges of studying metabolic changes during *in vivo* human thymocyte development, *in vitro* models such as the ATO are potentially more tractable to perform metabolic measurements and perturbations. However, studies have not yet probed how well *in vitro* models reflect *in vivo* thymocyte metabolic activity.

This report uses transcriptomics and extracellular flux analyses to metabolically profile human and murine thymocytes ranging from early DN to mature SP stages. We also compare the metabolic profiles of primary thymic populations to those of *in vitro* ATO-derived populations from the DN to DP stages. Our study thus uncovers fundamental metabolic patterns in T cell development that are conserved in human and murine thymus, as well as *in vitro* culture conditions.

## Materials and Methods

### Ethics Statement

All bone marrow and thymus harvesting from mice were conducted according to the National Institutes of Health Guide for Care and Use of Experimental Animals under a protocol approved by the UCLA Chancellor’s Animal Research Committee (ARC).

Cord blood and thymus samples were acquired through the UCLA Translational Pathology Core Laboratory (TPCL) and Children’s Hospital Los Angeles, respectively. As all samples were considered waste tissue without patient identifiers, the Institutional Review Board (IRB) deemed this work as not human subject research.

### Mice

All animal experiments were conducted under a protocol approved by the UCLA Chancellor’s ARC. This study used 1-4 month-old mice from Jackson Laboratory (Bar Harbor, Maine): C57BL/6 [(Cat# JAX:000664) mice and RAG1^-/-^ mice (JAX:002216)]. Mice from both sexes were randomly allocated to experimental groups.

### Cell Lines

The generation of MS5-mDLL4 and MS5-hDLL4 cell lines in our lab has been previously described (27, 29).

### Isolation of Murine Bone Marrow HSPCs for M-ATO Culture

Fresh or frozen bone marrow cells were enriched for HSPCs by negative cells selection of Lin- cells by magnetic cell sorting (MACS) using Murine Lin depletion Kit (Miltenyi, Auburn CA, Cat# 130-110-470). HSPCs were isolated by FACS sorting using following phenotype: LSK (Lin^-^ Sca1^+^ cKit^+^; Lin^-^ stands for: Ter119^-^, TCRγδ^-^, B220^-^, CD19^-^, CD11c^-^, CD11b^-^, Gr1^-^, NK1.1^-^, CD5^-^, CD4^-^, CD8^-^, CD3^-^). Sorted cells were immediately seeded into MS5-mDLL4 M-ATOs.

### Murine Artificial Thymic Organoid (M-ATO) Cultures

M-ATOs were generated as previously described (27). MS5-mDLL4 cells were harvested by trypsinization and resuspended in serum free M-ATO culture medium (“D/F12-B27”) composed of DMEM-F12 (Gibco, Cat# 11320033), 2% B27 supplement (ThermoFisher Scientific, Grand Island, NY, Cat# 17504-044), 30 µM L-ascorbic acid 2-phosphate sesquimagnesium salt hydrate (Sigma-Aldrich, St. Louis, MO, Cat# A8960-5G) reconstituted in 1X PBS, 1% penicillin/streptomycin (Gemini Bio-Products, West Sacramento, CA, Cat# 400-109), 1% Glutamax (ThermoFisher Scientific, Grand Island, NY, Cat# 35050-061), 5 ng/ml rmFLT3L (Peprotech, Rocky Hill, NJ, Cat# 250-31L), 5 ng/ml rmIL-7 (Peprotech, Cat# 217-17), 10 ng/ml rmSCF (Peprotech, Cat# 250-03) (of note SCF was added only for the first week of culture) and beta mercaptoethanol (bME) (0.05mM) (Sigma-Aldrich, Cat# M7522). D/F12-B27 was made fresh weekly. 1.5x10^5^ MS5-mDLL4 cells were combined with purified murine HSPCs (100-4000 cells/ATO). M-ATOs were plated on a 0.4 µm Millicell transwell insert (EMD Millipore, Billerica, MA; Cat. PICM0RG50) placed in a 6-well plate containing 1 ml D/F12-B27 per well. Medium was changed completely every 3-4 days by aspiration from around the cell insert followed by replacement with 1 ml with fresh D/F12 and cytokines.

### Isolation of Murine Thymocytes and M-ATO-Derived Cells

Thymic fragments from the mouse thymus were finely dissected in FACS buffer (1X PBS/0.5% bovine serum album/2mM EDTA) and disrupted by pipetting to release thymocytes into suspension, followed by passage through a 70 µm nylon strainer. Cells were then stained for flow cytometry.

At the indicated times, M-ATO-derived T cells were harvested by adding FACS buffer (1X PBS/0.5% bovine serum album/2mM EDTA) to each cell insert and briefly disaggregating the M-ATO by pipetting with a 1 ml “P1000” pipet, followed by passage through a 70 µm nylon strainer. Cells were then stained for flow cytometry.

For Seahorse Extracellular Flux Analyses, thymocytes from mouse thymus and M-ATO were enriched for DN, ISP8, and SP8 cells by negative selection of CD4^-^ cells, whereas DP early and SP4 cells were enriched in the CD4^+^ cell fraction, using magnetic cell sorting (MACS) with PE-CD4 (RM4-4) antibody and Anti-PE Microbeads Kit (Miltenyi, Auburn CA, Cat: #130-048-801).

Flow cytometry cell sorting of mouse thymic and M-ATO-derived T cell populations used surface phenotypes as detailed in **Supplementary Table 1**.

### Isolation of Human CD34^+^CD3^-^ HSPCs

Neonatal umbilical cord blood was obtained from discarded cord and placental units from deliveries at UCLA. All tissue samples were obtained under UCLA Institutional Review Board (IRB)-approved protocols or exemptions. All samples were enriched for mononuclear cells by Ficoll-Paque (GE Healthcare Life Sciences, Pitssburgh, PA) gradient centrifugation followed by positive selection of CD34^+^ cells by magnetic cell sorting (MACS) using the CD34 MicroBead Kit UltraPure (Miltenyi Biotec, Auburn, CA). After MACS, CD34^+^-cell-enriched fractions were utilized immediately or cryopreserved. Prior to use, cryopreserved cells were thawed, and the residual T cells were depleted by FACS by sorting for CD34^+^CD3^-^ cells, which were immediately seeded into H-ATOs.

### Human Cord Blood Artificial Thymic Organoid (H-ATO) Cultures

Generation of H-ATO cells were performed as previously described (28, 29), with the exception of the recombinant human IL-7 concentration in culture medium. MS5-hDLL4 cells were harvested and resuspended in serum-free H-ATO culture medium (‘RB27’), which was composed of RPMI 1640 (Corning, Manassas, VA), 4% B27 supplement (ThermoFisher Scientific, Grand Island, NY), 30 M l-ascorbic acid 2-phosphate sesquimagnesium salt hydrate (Sigma-Aldrich, St. Louis, MO) reconstituted in 1X PBS, 1% penicillin–streptomycin (Gemini Bio-Products, West Sacramento, CA), 1-2% Glutamax (ThermoFisher Scientific, Grand Island, NY), 5 ng/ml recombinant human FLT3 ligand (rhFLT3L) and 2.5-5 ng/ml recombinant human IL-7 (rhIL-7) (Peprotech, Rocky Hill, NJ). RB27 was made fresh weekly. 1.5 x 10^5^ MS5-hDLL4 cells were combined with 1 x10^2^ to 1 x10^5^ purified CD34^+^CD3^-^ cells per H-ATO. For each H-ATO, a 0.4-m Millicell Transwell insert (EMD Millipore, Billerica, MA; Cat. PICM0RG50) was placed in a 6-well plate containing 1 ml RB27 per well. Medium was changed completely every 3–4 d by aspiration from around the cell insert followed by replacement with 1 ml fresh RB27 and cytokines. H-ATOs were cultured for up to 10 weeks.

### Isolation of Human Thymocytes and H-ATO-Derived Cells

Postnatal human thymi were obtained under an IRB exemption as discarded waste from patients undergoing cardiac surgery at Children’s Hospital Los Angeles (CHLA). Thymic fragments were finely dissected in RPMI medium (Cellgro) and disrupted by pipetting to release thymocytes into suspension, followed by passage through a 70-µm nylon strainer. Cells were analyzed fresh on the same or the following day.

At the indicated times, H-ATO cells were harvested by adding FACS buffer (0.5% BSA and 2 mM EDTA in 1X PBS) to each well and briefly disaggregating the H-ATO by pipetting with a 1 ml ‘P1000’ pipet tip, followed by passage through a 50-m nylon strainer.

For Seahorse Extracellular Flux Analyses, thymocytes from human thymus or human ATO were enriched for DN, ISP4, and SP4 cells by negative selection of CD8^-^ cells, or enriched for DN and SP8 cells by negative selection of CD4^-^ cells using magnetic cell sorting (MACS) with PE-CD8 (HIT8a) or PE-CD4 (OKT4) antibody respectively and Anti-PE Microbeads UltraPure Kit (Miltenyi, Auburn CA, Cat: #130-105-639).

Flow cytometry cell sorting of human thymic and H-ATO-derived T cell populations used the surface phenotypes as detailed in **Supplementary Table 1**.

### Flow Cytometry

All flow cytometry stains were performed in 1X PBS/0.5% BSA/2 mM EDTA for 20 min on ice. TruStain FcX (Biolegend, San Diego, CA, Cat#101320 for mouse, Cat#422302 for human) was added to all murine samples for 5 min prior to antibody staining. DAPI (Life technologies, Carlsbad, CA, Cat# D1306) was added to all samples prior to analysis.

Analysis was performed on an LSRII Fortessa, and FACS sorting on FACSARIA or FACSARIA-H instruments (BD Biosciences, San Jose, CA) at the UCLA Broad Stem Cell Research Center Flow Cytometry Core.

For all analyses DAPI^+^ cells were gated out, and single cells were gated based on FSC-H *vs.* FSC-W and SSC-H *vs.* SSC-W. Gating strategies were performed as detailed in **Supplementary Table 1** and presented in previous reports (27, 28).

Anti-mouse and anti-human antibody clones used for surface and intracellular staining were obtained from Biolegend (San Diego, CA) or BD Bioscience (San Jose, CA), and are listed in Key Resource Table in **Supplementary Table 4**.

For measurement of mitochondrial mass, thymocytes were stained with surface markers, washed in MB, and incubated in medium containing 10 nM MitoTracker Green (Life Technologies, Carlsbad, CA, Cat# M7514) for 30 min at 37°C.

For apoptosis measurements, thymocytes were stained with surface markers, washed 2x with cold PBS, resuspended in Annexin V binding buffer (Biolegend, San Diego, CA, Cat# 422201), and incubated with Annexin V (Biolegend, San Diego, CA, USA, Cat# 640945) and DAPI (Life technologies, Carlsbad, CA, Cat# D1306) for 15 min at RT in the dark. Cells were analyzed within 1 hour of Annexin V staining.

Flow cytometry data were analyzed with FlowJo software (Tree Star Inc.).

### Extracellular Flux Analysis

Thymocytes were centrifuged onto a XF96 well plate coated with Poly-D-Lysine and washed twice with DMEM assay medium containing 5 mM glucose, 2 mM L-glutamine, 1 mM sodium pyruvate, and 5mM HEPES. OCR and ECAR were measured under basal conditions and in response to 2 µM oligomycin, two injections of 0.75 µM fluoro-carbonyl cyanide phenylhydrazone (FCCP), and 2 µM Antimycin/Rotenone using the XF-96 Extracellular Flux Analyzer (Seahorse Bioscience). Extracellular flux rates were normalized to cell number in each well using the Wave software (**Agilent**). Raw data were imported into R to calculate extracellular flux measurements: basal ECAR = baseline ECAR; basal OCR = baseline OCR – Rot/AA OCR; maximal OCR = maximum OCR – Rot/AA OCR; spare respiratory capacity = maximal OCR – basal OCR; OCR/ECAR ratio = basal OCR/basal ECAR) (30).

### Quantification and Statistical Analysis

In all figures, *n* represents independent experiments and data are represented as mean ± standard deviation (s.d.). Statistical analysis was performed using R or GraphPad Prism software, *p*-values were calculated from the two-tailed unpaired *t* test or multiple t-test, adjusted *p*-values were calculated using the Benjamini-Hochberg method. The *p*-values are directly indicated on the figure, above the corresponding graphs. * or # *p*< 0.05; ** or ## *p*< 0.01; and *** or ### *p*< 0.001 were considered statistically significant.

### Correlation Analysis

Extracellular flux data was asinh transformed with a cofactor of 5. RNA-seq data were count-based normalized and variance-stabilized as described below, and *Z*-score normalization was performed on genes from specified metabolic pathways in each compartment. To represent gene expression in the DN compartment, the weighted average of DN1-3 or Thy1-3 gene expression were calculated according to the proportion of each population in at least 3 experimental samples. The following weighted averages were used. Mouse thymus: DN1 (4%), DN2 (3%), DN3 (93%). M-ATO: DN1 (1%), DN2 (1%), DN3 (98%). Human thymus: Thy1 (0.1%), Thy2 (59.9%), Thy3 (40%). Human ATO: Thy1 (0.1%), Thy2 (79.9%), Thy3 (20%). Correlation between extracellular flux and metabolic gene expression data across all thymocyte populations (DN, ISP, SP8, and SP4 in M- and H-THY; or DN, ISP, and DP early in M- and H-ATO) were calculated using the ggcorr R package. Adjusted *p*-values for each Pearson correlation coefficient were calculated using the Benjamini-Hochberg method, and plots were created using the ggplot2 R package.

### RNA Sequencing and Data Analysis

The preparation and analysis of mouse thymus and M-ATO RNA-seq libraries for thymic subsets have been previously described and raw sequence files are available at NCBI’s GEO (GSE146224) (27). For the new human thymus and H-ATO libraries reported here, RNA was extracted from each of the indicated human thymic or H-ATO-derived populations isolated by FACS, as described above and in **Supplementary Table 1**. Total RNA was isolated using the RNeasy Micro kit (Qiagen). Sequencing libraries were prepared with the Ovation RNA-seq system V2 kit (NuGEN). Paired-end 150 bp sequencing was performed on the Illumina HiSeq 4000.

The STAR ultrafast universal RNA-seq aligner v2.7.0d (31) was used to align reads to a genome index including both the genome sequence (GRC38 primary assembly) and the comprehensive genome annotation from Gencode (version 36). Alignment files were used to generate strand-specific, gene-level count summaries with STAR’s built-in gene counter. Expression estimates provided were computed in units of fragments per kilobase of mappable length and million counts (FPKMs). Count-based normalized and variance-stabilized data (32) was used for all ordination, differential and clustering analyses, and all figures unless otherwise noted. Principal component analysis (PCA) was performed with the function prcomp in R (https://www.R-project.org/) using standardized data as input. To facilitate the integration of the datasets, standardization was performed independently prior to PCA.

**Figure 1:**
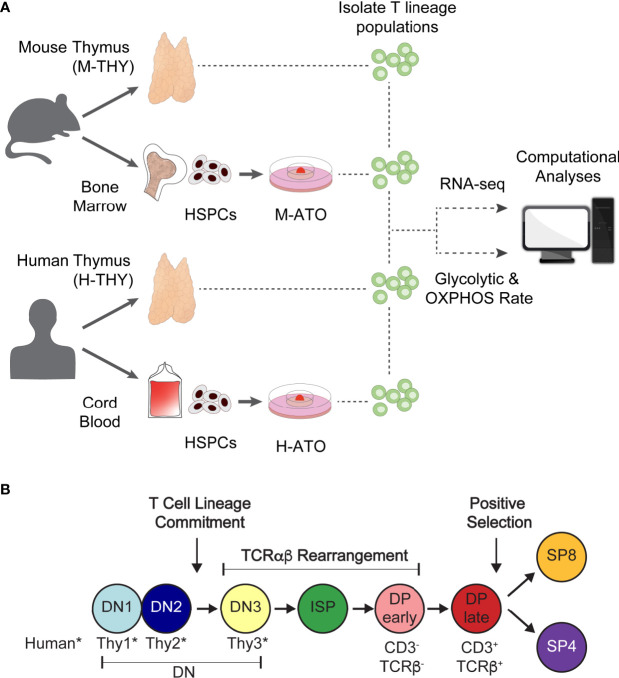
**(A)** Experimental schema for metabolic analysis of murine and human T cell development in primary thymus and *in vitro* ATO cultures. HSPCs isolated from mouse bone marrow or human cord blood were seeded in M-ATO and H-ATO systems respectively. T lineage populations isolated from each tissue were subject to RNA-seq or extracellular flux measurements to determine glycolytic and respiration rates (Seahorse Assay). Metabolic transcriptional and flux results were integrated to determine statistical correlation between datasets. **(B)** Schematic of T lineage populations isolated for RNA-seq analyses or Seahorse Assays. Detailed population phenotypes for isolation are provided in **Supplementary Table 1**. (*DN populations are named DN1-3 in mouse, and Thy1-3 in human).

Differential expression analyses was performed with DESeq2 (Bioconductor, v3.7, RRID : SCR_015687) (32). We performed pair-wise comparisons between sequential thymocyte populations within human thymic or H-ATO compartments, or between analogous human thymic and H-ATO-derived populations. We defined a set of 769 variable genes (**Supplementary Table 2**) for further analyses as: fold-change greater than 2, Benjamini-Hochberg adjusted Wald test *p*-value less than 0.01 in at least one pair-wise test, and a minimum expression of 5 FPKMs in at least one sample; genes with less than half-count per million in all samples, count outliers, or low mappability (<50bp) were filtered out from the differential gene expression analysis (12, 27, 32). This set of most significantly variable genes was subjected to model-based clustering using MBCluster.Seq (33) to classify them based on their overall abundance profile across populations (**Figure 1B**). We set the starting number of clusters to 100, and then manually merged them to generate a set of 16 non-redundant gene classes.

Functional enrichment for genes selected in the tests and clusters above was performed with Metascape (34). All heatmap visualizations were created using pheatmap R package, and all line plots were created using spline function and ggplot2 package in R.

## Results

### Experimental Approach for Metabolic Profiling of Murine and Human T Cell Differentiation

To investigate the metabolic landscape of thymic T cell differentiation in murine and human systems, we followed the workflow presented in **Figure 1A**. We acquired transcriptional datasets in analogous thymocyte populations from murine thymus (M-THY hereafter), human thymus (H-THY hereafter), and *in vitro* systems. To generate *in vitro* thymocytes, HSPCs isolated from mouse bone marrow or human cord blood were seeded in mouse ATO (M-ATO hereafter) and human ATO (H-ATO hereafter) systems respectively (27, 28). Thymocytes were isolated from primary thymus or ATO-derived cultures and subjected to bulk RNA-sequencing, or extracellular flux measurements that reflect glycolytic and oxidative phosphorylation (OXPHOS) activity. Thymocyte developmental stages included in the analyses are shown (**Figure 1B** and **Supplementary Table 1**). As the SP4 population is scarce in H-ATOs, these were not collected for transcriptional analysis.

### Transcriptional Analysis of Primary Human Thymus and H-ATO

We recently reported that transcriptional regulation in M-ATO-derived thymocytes recapitulates T cell developmental programs in mouse thymus (27). Here, we similarly examined transcriptional profiles of phenotypically identical populations from primary human thymus and H-ATO.

**Figure 2:**
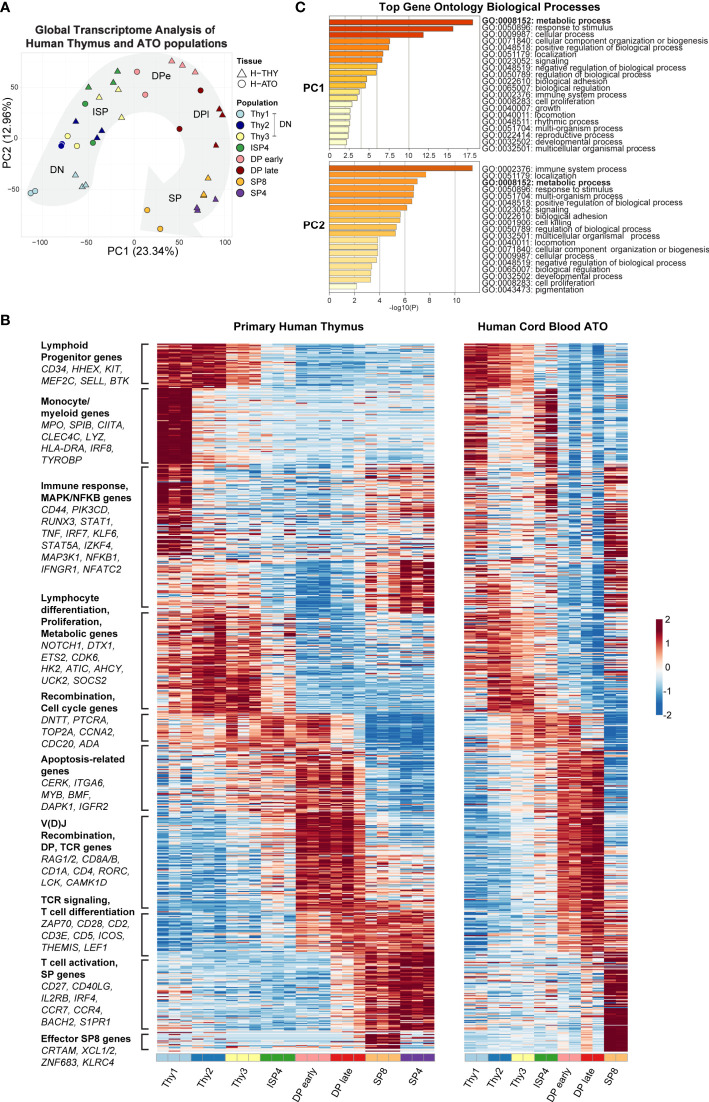
**(A)** Principal-component analysis (PCA) of gene expression for human thymic and H-ATO-derived populations. PC1 and PC2 are shown along with the percentage of gene expression variance explained. Clustering was obtained with data from all detected genes without additional filters. Population phenotypes from each source are provided in **Supplementary Table 1**. SP4 population was not collected from H-ATO. Background arrow shows direction of differentiation. (n=3 independent replicates for H-THY; n=2 for H-ATO) **(B)** Hierarchical model-based clustering of 769 highly variable genes classified as differentially expressed (Wald adjusted p value < 0.01, fold change > 4) within and between human thymic and ATO-derived populations. The x axis indicates populations isolated for analysis. Each heatmap represents *z*-scores of normalized variance-stabilized gene expression data. To summarize the unsupervised hierarchical clustering results, we highlight for each cluster an ontology annotation significantly associated with genes overrepresented in each cluster, along with manually selected genes known to be involved in T cell development. The full list of genes as ordered in the heatmap is provided in **Supplementary Table 2**. (n=3 independent replicates for H-THY; n=2 for H-ATO). **(C)** Top GO terms significantly enriched in PC1 and PC2.

Principal-component analysis (PCA) using global genome-wide expression profiles showed that H-THY and H-ATO-derived populations overall exhibited a similar progression along the first and second principal components (PC1: 23.34% variance; PC2: 12.96% variance) (**Figure 2A**).

Next, we performed unsupervised hierarchical model-based clustering on human thymic and ATO-derived populations. Due to differences in baseline gene expression, we first normalized gene expression data and then classified genes by their overall expression profile using an unsupervised approach and stringent thresholds. We used pairwise statistical tests between sequential populations within each sample tissue (e.g., H-THY ISP4 *versus* H-THY DP early), or between equivalent populations in thymic and ATO tissues (H-THY SP8 *versus* H-ATO SP8), to identify a set of most variable genes (769 human genes) that were then subject to hierarchical model-based clustering (provided in **Supplementary Table 2**). In a summary heatmap shown in **Figure 2B**, we reordered each cluster of differentially expressed genes to match peak expression with developmental stage (DN to SPs). We included annotations and representative genes for each cluster, as well as manually selected genes known to be involved in T cell development based on our previous transcriptional analyses in murine thymocytes (27). Human thymic and ATO gene expression patterns were highly similar overall and featured key markers of T cell development.

Gene ontology (GO) enrichment analysis of the top 500 ranked genes in PC1 and PC2 revealed that the category of metabolic processes was highly enriched in both PC1 and PC2 (**Figure 2C**). Several metabolic genes were also classified as being differentially expressed during development and clustered together, e.g., *HK2, ATIC, AHCY*, and *UCK2* (**Figure 2B**). Thus, metabolic gene signatures are a major component of the transcriptional differences between human thymocyte stages.

### Transcription of Core Metabolic Pathways in Primary Murine and Human Thymocytes

**Figure 3:**
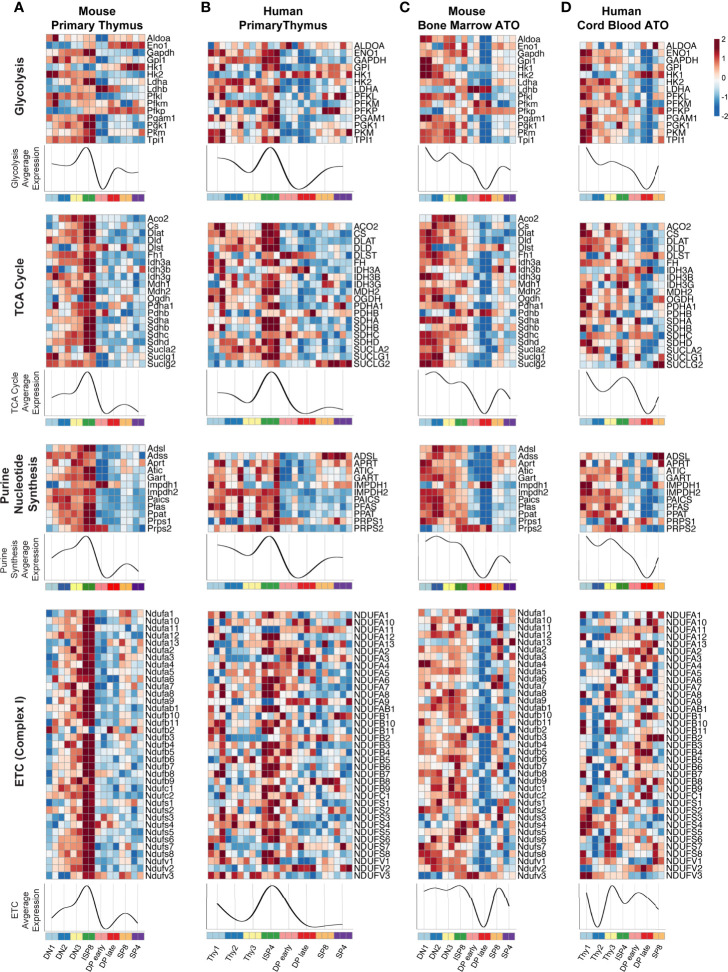
**(A–D)** Heatmaps of metabolic gene expression patterns in **(A)** primary mouse thymocytes (M-THY), **(B)** primary human thymocytes (H-THY), **(C)** mouse bone marrow ATO (M-ATO), and **(D)** human cord blood ATO (H-ATO). Major metabolic pathways are displayed: Glycolysis, nucleotide synthesis, TCA cycle, and ETC (*only de novo purine synthesis and an abbreviated ETC heatmap for Complex I is shown, additional nucleotide synthesis pathways and ETC complexes are included in* **Supplementary Figure 1**). Each individual heatmap represents *z*-scores of normalized variance-stabilized gene expression data. Line plots represent the average *z*-score for all genes in the heatmap. The x axis indicates populations isolated for analysis. SP8 and SP4 are distinct lineage fates but are represented in a linear progression for visualization purposes. (n=2 independent replicates per population in M-THY; n=3 in H-THY; n=2 in M-ATO; n=2 in H-ATO).

As global transcriptional analyses suggested that significant metabolic changes occur during thymopoiesis, we next performed a supervised analysis using a manually curated list of genes originally derived from the KEGG database. We focused on genes encoding enzymes directly involved in major oxidative and biosynthetic pathways, particularly glycolysis, *de novo* purine nucleotide synthesis, tricarboxylic acid cycle (TCA), and the electron transport chain (ETC) (**Figure 3**). We also analyzed other essential metabolic pathways, including fatty acid beta-oxidation (FAO), pentose phosphate pathway (PPP), *de novo* pyrimidine nucleotide synthesis, and the purine salvage pathway (**Supplementary Figure 1**). In both primary murine and human thymus, the expression of most glycolytic and TCA cycle genes, such as *LDHA, HK2, GAPDH, PGAM1, TPI1, CS, IDH3G*, and *PDHA1* were high at the DN1-DN3 or Thy1-3 stages (**Figures 3A, B**). After a marked peak at the ISP stage, transcription dramatically declined in the DP thymocytes. The subsequent SP stages showed some recovery in the transcription of glycolytic genes. Notably, enzyme isoforms in the glycolytic pathway displayed reciprocal gene expression at the ISP-DP transition: *Ldhb vs Ldha* (M-THY), *Pfkp vs Pfkl* (M-THY), *HK1 vs HK2* (M-THY and H-THY), *PFKP vs PFKM* (H-THY). A few differences between mouse and human thymus were also seen; for instance, *ENO1* is expressed most highly in the DN-ISP stages in human thymocytes but is expressed most highly in SP cells in mouse thymocytes.

Overall, genes within the *de novo* purine synthesis, and ETC pathways showed a similar pattern in primary thymocytes as seen for the glycolytic and TCA cycle genes (**Figures 3A, B** and **Supplementary Figures 1A, B**). FAO, PPP, *de novo* pyrimidine synthesis, and purine salvage pathways also showed a marked fall in transcription at the DP stages in primary thymus samples (**Supplementary Figures 1A, B**). Thus, murine and human thymocytes isolated from primary thymus shared a highly conserved pattern of core metabolic programs at distinct stages of T cell development, with a peak in transcription during the transitional ISP stage and a dramatic fall in the early (TCR^-^) and late (TCR^+^) DP stages.

### Transcriptional Pattern of Core Metabolic Programs During Murine and Human *In Vitro* Thymopoiesis

Next, we examined metabolic transcriptional patterns in murine and human *in vitro* thymopoiesis using the ATO system. Similar to the primary thymocytes in M-THY and H-THY, coordinated metabolic gene expression patterns were seen in the M-ATO and H-ATO: glycolytic and TCA cycle gene expression, including that of *LDHA, HK2, GAPDH, PGAM1, TPI1, CS, IDH3G*, and *PDHA1* was highest in DN-ISP stages, decreased at the DP stages, and recovered at the SP stages (**Figures 3C, D**). Enzyme isoform switching was again seen in the M-ATO and H-ATO at the ISP-DP transition in *Ldhb vs Ldha* (M-ATO), *Pfkp vs Pfkl* (M-ATO), and *HK1 vs HK2* (H-ATO). Gene expression in the nucleotide synthesis, FAO, and PPP pathways was also lowest at the DP stages in both ATO systems as well. (**Figures 3C, D** and **Supplementary Figures 1C, D**). A subset of metabolic genes showed an inverse pattern, e.g., *DLST* (TCA) and *ADA* (purine salvage) were upregulated at the DP stage across all conditions (in both species, *in vivo*, and *in vitro*) (**Figure 3** and **Supplementary Figure 1**).

Although metabolic gene expression was largely conserved across *in vivo* and *in vitro* conditions, some differences were noted. In contrast to DN1 cells in primary mouse thymus, DN1 cells in the M-ATOs exhibited high metabolic gene expression levels that were sustained rather than peaking at the ISP8 stage; moreover, the sharp downregulation of metabolic gene expression seen in the M-THY was delayed in the M-ATO, with the nadir observed at the DP late instead of DP early stage (**Figures 3A, C** and **Supplementary Figures 1A, C**). These subtle differences were also observed in the H-ATO. However, the starkest difference between H-ATO and H-THY samples was the uncoordinated expression of mitochondrial ETC genes in the H-ATO (**Figures 3B, D** and **Supplementary Figures 1B, D**).

### Metabolic Extracellular Flux Dynamics Are Conserved in Murine and Human Thymocytes

**Figure 4:**
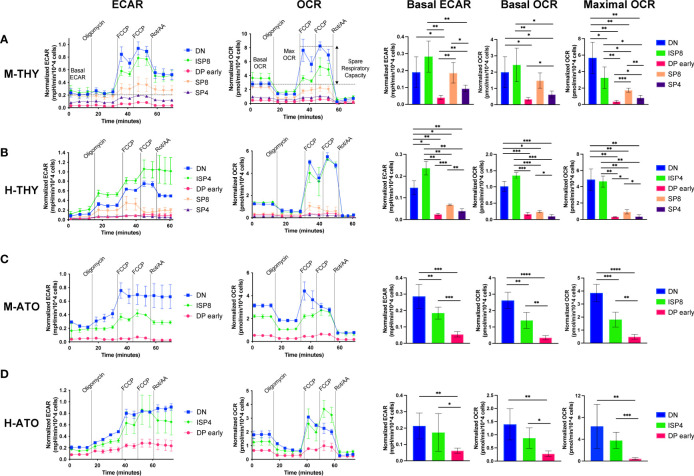
**(A–D)** Extracellular flux analyses of thymocyte populations in **(A)** M-THY, **(B)** M-ATO, **(C)** H-THY, and **(D)** H-ATO. ECAR (left) and OCR (right) measurements are displayed for each time point over the course of several pharmacologic agents: oligomycin, FCCP, and Rotenone/Antimycin (Rot/AA). Line plots on the left show one representative experiment out of n=6 independent experiments in M-THY; n=6 M-ATO; n=3 H-THY (except for ISP4: n=2); n=6 H-ATO (except for ISP4: n=5). Symbols and error bars represent mean +/- sd for 2-4 technical replicates (wells). Measurements are normalized to cell number per well. The bar graphs on the right consist of aggregated data from all experiments. Basal ECAR, basal OCR, and maximal OCR for each population were calculated as detailed in the methods and compared. (error bars: sd, significance: multiple t-test p values *p < 0.05; **p < 0.01, ***P < 0.001, ****P < 0.0001).

To investigate whether metabolic gene transcription patterns reflect bioenergetic changes during T cell development, we assessed metabolic extracellular flux data in developing T cells from primary human thymus and murine thymus (**Figures 4A, B**). We measured extracellular acidification rate (ECAR) and oxygen consumption rate (OCR), indicators of glycolysis and oxidative phosphorylation (OXPHOS) respectively, at basal conditions and after addition of pharmacological agents. When the drug FCCP is added, oxygen consumption is uncoupled from ATP synthesis and cells are induced to utilize their maximal respiratory capacity. Given the RNA-seq results, we chose to compare total DN, ISP, and the subset of DPs that do not yet express cell surface TCR (“DP early”), as well as SP4 and SP8 cells.

In primary murine thymus, DN and ISP8 cells had higher basal ECAR and OCR than DP and SP cells (**Figure 4A**). DN cells exhibited a robust response to mitochondrial uncoupling with high maximal OCR and spare respiratory capacity (SRC) (**Figure 4A** and **Supplementary Figure 2A**). In contrast, DP early cells showed the lowest basal ECAR and OCR, and essentially no change between basal and maximal OCR in response to FCCP stimulation. ISP8 cells had similar basal ECAR and OCR to DN cells but were less able to respond to FCCP addition compared to DN cells.

The pattern of metabolic flux was similar in human thymus, with DN and ISP4 cells showing high basal ECAR, basal OCR, maximal OCR, and SRC (**Figure 4B** and **Supplementary Figure 2B**). Again, DP cells showed low basal ECAR and OCR, and almost no difference between OCR at baseline and in response to FCCP stimulation. Interestingly, in murine and human primary thymus, there was higher glycolytic and oxidative phosphorylation activity in SP8 cells compared to SP4 cells, as shown by significantly higher basal ECAR and OCR (**Figures 4A, B**). Thus, as suggested by transcriptional profiles in primary human and murine thymocytes, metabolic activity is highest in the early DN-ISP populations, drops significantly at the DP early stage, and shows some recovery in the SP populations.

### Metabolic Flux Dynamics in Thymocytes Generated *In Vitro*

We next measured glycolytic activity and mitochondrial respiration in the DN, ISP, and DP early populations generated *in vitro* from M-ATO and H-ATO. SP populations from the ATO were not included due to the high cell numbers (>300,000 cells) required for robust extracellular flux measurements per experiment. In the M-ATO, DN cells had the highest basal ECAR and OCR, as well as the strongest response to FCCP addition with high maximal OCR and SRC (**Figure 4C** and **Supplementary Figure 2C**). Basal ECAR and OCR in ISP8 cells were higher than that of DP early cells but lower than that of DN cells; moreover, ISP8 cells were less able than DN cells to respond to mitochondrial uncoupling. DP early cells showed the lowest basal ECAR and OCR, and very little difference between basal and maximal OCR.

Similar to M-ATOs, DN and ISP4 cells from H-ATOs exhibited high basal ECAR and OCR, and a robust response to FCCP stimulation producing high maximal OCR and SRC (**Figure 4D** and **Supplementary Figure 2D**). DP early cells showed the lowest basal ECAR and OCR, and essentially no difference in baseline and maximal OCR.

No major differences in OCR and ECAR results were seen when comparing M-THY and M-ATO thymocytes, other than a lower SRC in DN cells from M-ATO compared to those from M-THY (**Supplementary Figure 2E**). H-THY and H-ATO thymocytes also showed comparable OCR and ECAR results, except that basal ECAR was higher in DP early cells from H-ATO than that from H-THY (**Supplementary Figure 2F**).

We also quantified mitochondrial mass using MitoTracker Green (MTG) dye in primary and ATO-derived thymocytes. MTG dye measurements were not significantly different between M-THY and M-ATO thymocytes, and our results were reminiscent of prior studies on mitochondrial mass in primary mouse thymocytes (**Supplementary Figure 3A**) (20, 24, 35). H-THY and H-ATO thymocyte populations also incorporated comparable MTG levels, with the exception of the Thy3 stage (**Supplementary Figure 3B**). In both mouse and human systems, the earliest thymic progenitor populations DN1/Thy1 cells had low MTG, which is consistent with a previous report showing that HSCs express transporters that efflux mitochondrial dyes (36). While mitochondrial mass was relatively high in populations undergoing beta-selection (DN3 in mouse, ISP4 in human), mitochondrial mass in DP early cells did not seem to reflect the significant decrease in ETC gene expression in mouse or human thymocytes.

In addition, we performed Annexin V staining on primary mouse thymus and ATO thymocyte populations to identify early apoptotic cells (DAPI- Annexin V+) that might have been included in our data analyses, particularly of the DP early population. We did not see a significant difference in the proportion of early apoptotic cells in the ISP8 and DP early populations in either the M-THY or M-ATO, even though significant differences in glycolytic and mitochondrial metabolism are seen during this transition (**Supplementary Figure 3C**).

Overall, the ATO systems recapitulated the metabolic shift from high to low glycolytic and OXPHOS activity between the DN to DP stages observed in primary thymus. Moreover, despite differences in mitochondrial gene expression, mitochondrial dye content largely corresponds in H-THY and H-ATO thymocytes. Thus, *in vivo* and *in vitro* developing thymocytes reveal a strikingly conserved pattern in metabolic activity during thymopoiesis.

### Correlation Analyses of Metabolic Transcriptional and Bioenergetics Profiles in Thymus and ATO T Cell Development

**Figure 5:**
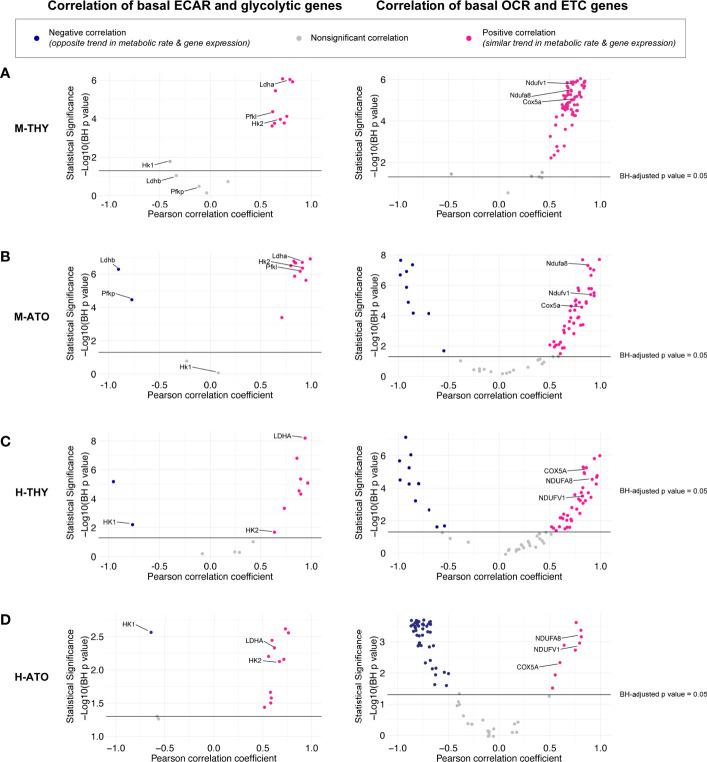
**(A–D)** Scatter plots show correlation between (left) glycolytic enzyme gene expression and basal glycolysis (ECAR), or (right) correlation between electron transport chain gene expression and basal respiration (OCR) in **(A)** M-THY, **(B)** M-ATO, and **(C)** H-THY, and **(D)** H-ATO. The x axis represents the Pearson correlation coefficient R values calculated for individual genes within the specified metabolic pathway for all cell populations for which respective extracellular flux data was collected. The y axis represents the statistical significance of each R value using -log10 of Benjamini-Hochberg adjusted p-values. Solid black line indicates an adjusted p-value of 0.05; all R values above the line are statistically significant. Genes with R values >0.5 (pink) are positively correlated with metabolic flux data, while genes with R values <-0.5 (blue) are negatively correlated with metabolic flux data. Nonsignificant or weak correlation R values >-.05 or <0.5 are indicated in grey. Certain genes of interest are indicated. (M-THY: n=6 flux analysis values for 5 populations, n=2 RNA-seq replicates; H-THY: n=3 flux analysis values for 5 populations, n=3 RNA-seq replicates; M-ATO: n=6 flux analysis values for 3 populations, n=2 RNA-seq replicates; H-ATO: n=6 flux analysis values for 3 populations, n=2 RNA-seq replicates).

It has been previously shown that trends in extracellular flux measurements can be associated with changes in the expression of specific enzymes in different cell types and states (30). Transcriptional and bioenergetics data appear to match in all systems except the ETC in the H-ATO. Thus, we performed further quantitative analyses to uncover the relationship between the two datasets. Correlation analyses between gene expression data and extracellular flux measurements were performed across thymocyte populations in M-THY, M-ATO, H-THY, or H-ATO (**Figure 1A**). Transcription levels of most glycolytic genes positively correlated with glycolytic flux results across all thymocyte sources (M-THY, M-ATO, H-THY, H-ATO), including *LDHA*, *HK2, PGAM1*, and *PGK1*; again, the few glycolytic genes that showed negative correlation with ECAR were isoforms noted earlier, e.g., *Ldhb* and *Pfkp* in M-ATO, and *HK1* in H-THY and H-ATO (**Figures 5A–D**, **Supplementary Table 3**).

In M-THY, M-ATO, and H-THY, RNA levels of most ETC pathway genes were positively correlated with mitochondrial respiration activity (**Figures 5A–C**). In keeping with the disorganized pattern of ETC gene expression seen in the H-ATOs (**Figure 3D** and **Supplementary Figure 1D**), there was an overall inverse correlation of transcription with mitochondrial respiration activity in this model (**Figure 5D**). Nonetheless, the ETC genes *COX5A, NDUFA8*, and *NDUFV1* were consistently positively correlated with OCR in all systems.

**Figure 6:**
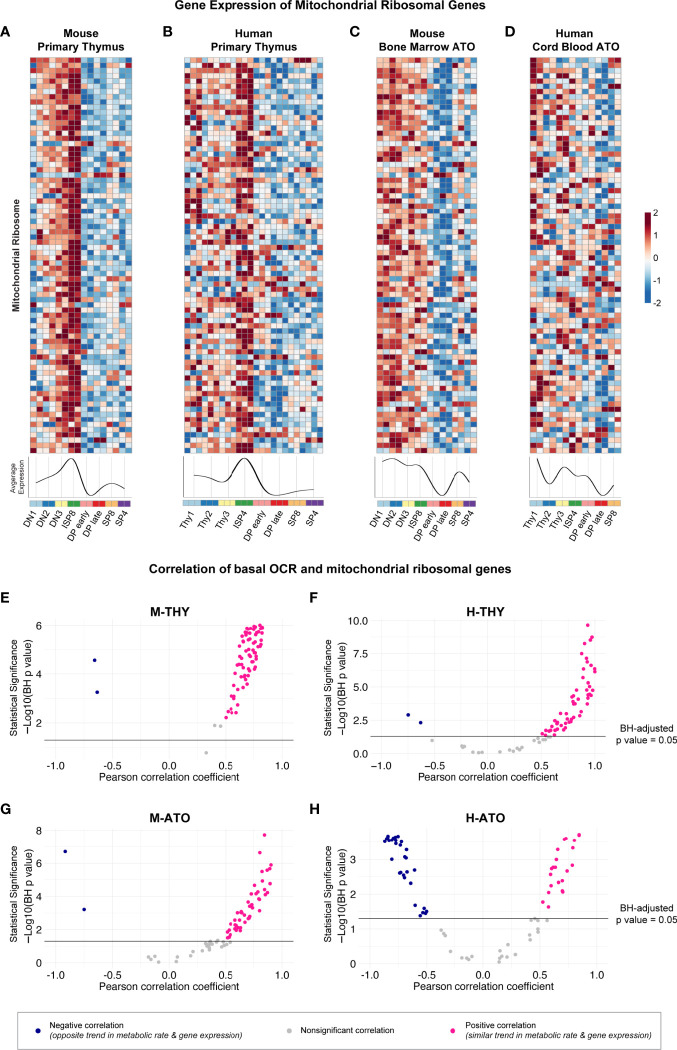
**(A-D)** Heatmaps and summary line plots of mitochondrial ribosomal genes in **(A)** M-THY, **(B)** H-THY, **(C)** M-ATO, and **(D)** H-ATO. The x axis represents cells sorted from progenitor (DN1 or Thy1) to mature single-positive (SP) T cells. Each individual heatmap represents *z*-scores of normalized variance-stabilized gene expression data. Line plots represent the average *z*-score for all genes in the heatmap. (n=2 independent replicates per population in M-THY; n=3 in H-THY; n=2 in M-ATO; n=2 in H-ATO). **(E–H)** Scatter plots show correlation between mitochondrial ribosomal genes and basal respiration (OCR) in **(E)** M-THY, **(F)** H-THY, **(G)** M-ATO, and **(H)** H-ATO. The x axis represents the Pearson correlation coefficient R values calculated for individual genes within the specified metabolic pathway for all thymocyte populations for which respective extracellular flux data was collected. The y axis represents the statistical significance of each R value using -log10 of Benjamini-Hochberg adjusted p-values. Solid black line indicates an adjusted p-value of 0.05; all R values above the line are statistically significant. Genes with R values >0.5 (pink) are positively correlated with metabolic flux data, while genes with R values <-0.5 (blue) are negatively correlated with metabolic flux data. Nonsignificant or weak correlation R values >-.05 or <0.5 are indicated in grey. (M-THY: n=6 flux analysis values for 5 populations, n=2 RNA-seq replicates; H-THY: n=3 flux analysis values for 5 populations, n=3 RNA-seq replicates; M-ATO: n=6 flux analysis values for 3 populations, n=2 RNA-seq replicates; H-ATO: n=6 flux analysis values for 3 populations, n=2 RNA-seq replicates).

Previously, the ImmGen report in murine thymus showed that the downregulation of ribosomal genes was associated with the decrease in metabolic genes at the DP stage (13). Therefore, we next probed whether structural ribosomal gene expression patterns strongly correlated with metabolic flux data. Mitochondrial ribosomal gene expression was the highest at the DN-ISP stages and lowest at the DP early-DP late stages in M-THY, H-THY, and M-ATO (**Figures 6A–C**). However, in the H-ATOs, the majority of mitochondrial ribosomal genes showed an uncoordinated expression pattern similar to that of the ETC genes (**Figures 6D**, **3D**). Correspondingly, mitochondrial ribosomal expression positively correlated with basal mitochondrial respiration rates in M-THY, H-THY, and M-ATO (**Figures 6E–G**), while most mitochondrial ribosomal genes in the H-ATO correlated inversely or not at all with basal OCR, as seen previously with the ETC genes (**Figures 6H**, **5D**). We also analyzed cytoplasmic ribosomal gene expression in H-THY and H-ATO, both of which showed global downregulation at the DP stages (**Supplementary Figures 4A, B**). In contrast to the mitochondrial ribosomal genes, cytoplasmic ribosomal gene expression positively correlated with basal glycolytic and respiration rates not only in the H-THY but also in the H-ATO (**Supplementary Figures 4C, D**).

Lastly, we assessed the gene expression of key signaling molecules and transcription factors that are known to regulate metabolic programs (5, 37–39), and analyzed how well their transcriptional pattern correlated with extracellular flux data (**Supplementary Figures 5A–H**). Since changes in basal ECAR and basal OCR were closely associated in thymocyte populations, we focused our analyses on basal OCR. First, we looked for metabolic regulators whose gene expression showed a consistently similar trend to basal OCR. In all systems, *NOTCH1* gene expression was strongly associated with basal mitochondrial respiration. Next, we examined key differences in metabolic regulator expression between primary thymus and the ATO systems. *STAT3*, *STAT5*, and *NFE2L2* were positively correlated with basal OCR in both the M-ATO and H-ATO but showed poor correlation in primary murine and human thymus. Most notably, whereas *MYC* gene expression was positively correlated with basal respiration in M-THY, H-THY, and M-ATO, *MYC* was inversely correlated with basal OCR in the H-ATO system. We carefully reviewed *MYC* expression in the H-ATO and observed peak expression in the Thy1 population, and then similar expression levels in the Thy2-3, ISP4, and DP early populations (**Supplementary Figure 5D**). The inverse correlation of *MYC* expression in the H-ATO was entirely driven by the persistence of high *MYC* expression in the DP early stage, even though DP early cells exhibit the lowest basal OCR (**Supplementary Figures 5D, H** and **Figure 4D**).

Thus, correlation analyses confirmed that metabolic transcriptional profiles reflected functional metabolic activity in both primary thymus and the ATO system; mitochondrial respiration in the human *in vitro* system (H-ATO) was the sole exception to this pattern.

### Thymocyte Metabolism in the Absence of TCR Rearrangement

**Figure 7:**
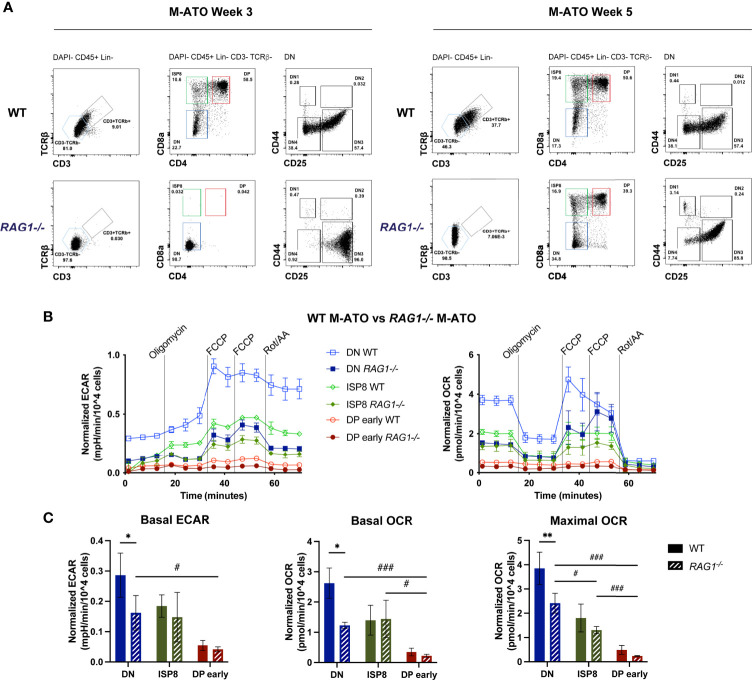
**(A)** Representative flow cytometry analysis of thymocyte populations from Week 3 and 5 M-ATOs derived from WT and *Rag1^-/-^* mouse bone marrow. **(B)** Extracellular flux analyses of thymocyte populations from Week 3 M-ATOs derived from WT or Week 5 M-ATOs derived from *Rag1^-/-^* mouse bone marrow HSPCs. **(C)** Comparison of basal ECAR, basal OCR, maximal OCR, between thymocyte populations from M-ATOs derived from WT or *Rag1^-/-^* bone marrow HSPCs. (WT: n=6, *Rag1^-/-^*: n=3 independent experiments). (error bars: sd, significance: multiple t-test p values, comparisons between WT and *Rag1^-/-^* ATO populations denoted by *; comparisons between *Rag1^-/-^* ATO populations denoted by . * or ^#^p < 0.05; ** or ^##^p < 0.01; *** or ^###^p < 0.001).

Since key metabolic transitions occur between stages undergoing TCRβ rearrangement (DN3 in mouse, ISP4 in human) and TCRα rearrangement (DP early) (15, 17, 18, 40), we tested the effect of removing TCR-dependent signaling on thymocyte metabolism using a *Rag1* (recombination activating gene 1)-deficient (*Rag1* ^-/-^) mouse model in the M-ATO (41). As expected, HSPCs from *Rag1* ^-/-^ marrow were unable to generate any CD3^+^TCRαβ ^+^ T cells in M-ATOs, and consistent with prior reports of *Rag1* ^-/-^ thymus, Week 3 M-ATO cultures did not progress past the DN stage (**Figure 7A**) (27). However, DPs have been shown to be able to develop in the *Rag1* ^-/-^ and *Rag2* ^-/-^ thymus through TCRβ chain-independent pathways and mutations resulting in activation of beta-catenin or Akt (42–44). Interestingly, CD3^-^TCRαβ^-^ DP cells have been reported to develop from human iPSCs derived from RAG-deficient patients in the OP9-DL system and PSC-ATO system, from RAG-deficient patient HSPCs in the H-ATO system, and from mouse HSPCs in late M-ATO cultures (27, 45–47). As mentioned previously, CD3^-^TCRαβ^-^ DPs were generated from *Rag1* ^-/-^ murine marrow by week 5 of M-ATO culture (27), allowing us to examine the metabolic flux of DP early cells and their precursors in the absence of TCR rearrangement (**Figure 7A**).

DN cells from *Rag1* ^-/-^ M-ATOs exhibited significantly decreased basal OCR and ECAR compared to that of WT DN cells (**Figures 7B, C**). Nonetheless, similar to WT M-ATOs, basal ECAR and basal OCR dropped significantly in DP early cells from *Rag1* ^-/-^ M-ATOs (**Figures 7B, C**). Thus, glycolytic and OXPHOS activity is at least partly dependent on *Rag1* activity in DN cells, but the metabolic shutdown at the DP early stage still occurs in the absence of TCR rearrangement.

## Discussion

**Figure 8:**
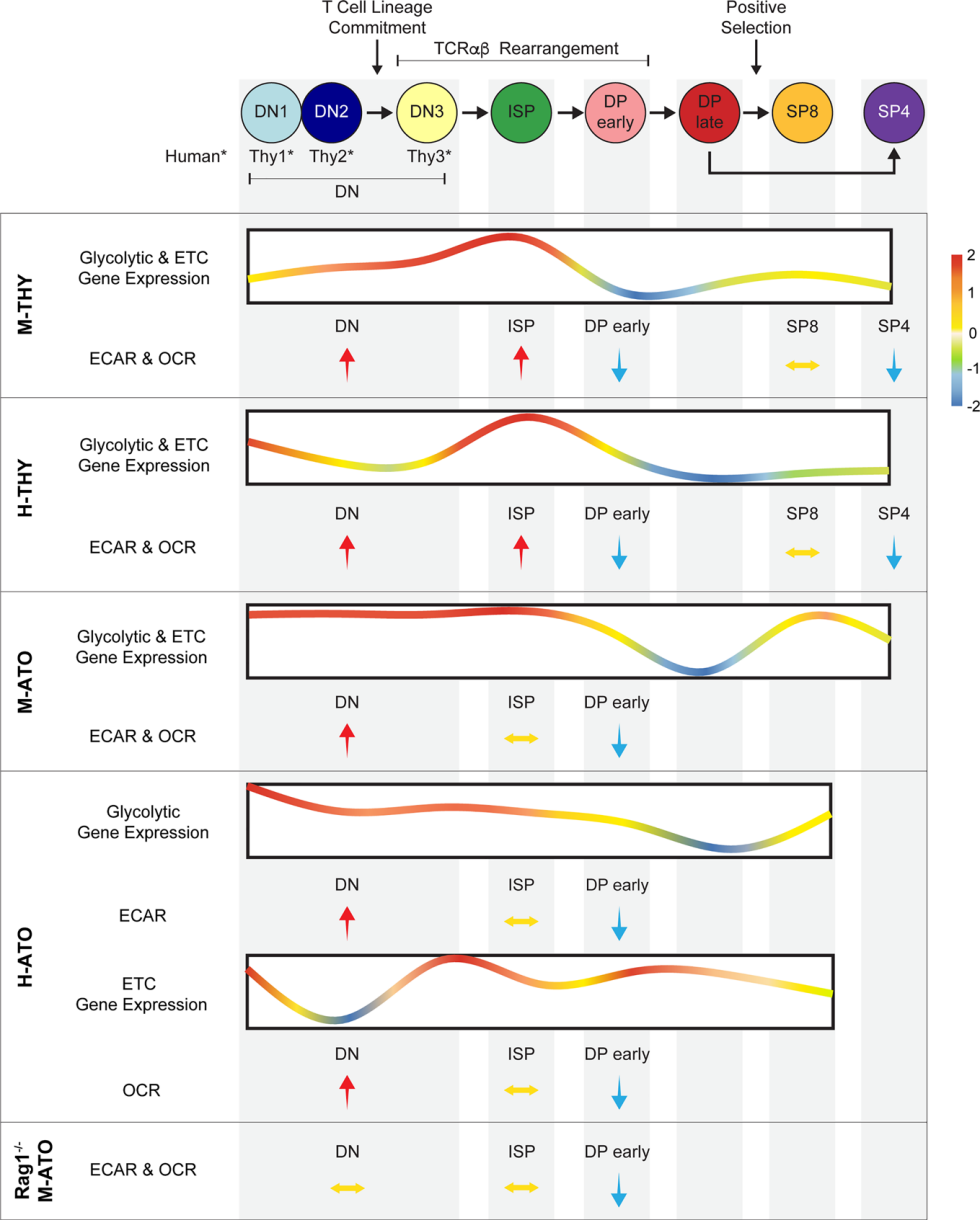
Schematic summarizing metabolism dynamics throughout thymocyte development in M-THY, H-THY, M-ATO, H-THY, H-ATO, and *RAG1^-/-^* M-ATO. Glycolytic and ETC gene expression patterns are represented with line plots. Color scale indicates relative fold change. Relative changes in extracellular flux between thymocyte populations are represented with arrows. H-ATO glycolytic and ETC results are shown separately. (*DN populations are named DN1-3 in mouse, and Thy1-3 in human.)

This report integrates comprehensive transcriptomics and extracellular flux data to present the first comparative analysis of metabolic dynamics during normal human and murine thymopoiesis. In addition, we show that metabolism is largely conserved during *in vitro* T cell development in which the complex multi-cellular and spatial organization of the thymus is absent. Previous studies have separately investigated microarray data in murine thymus (13), metabolic parameters in early murine thymocyte stages (20, 24), single cell RNA-seq in human thymus (48, 49), glucose and phosphate transporter (GLUT1, PiT1/2) expression in human thymocytes (50, 51), and metabolic parameters in activated human T cells (30, 52). Our combined analyses demonstrate that metabolic transcriptional profiles generally reflect functional metabolic activity and reveal remarkably conserved metabolic shifts during critical stages of T cell development (**Figure 8**). Thymocytes prior to the DP stage demonstrated peak gene expression levels of enzymes directly involved in glycolytic, TCA, and ETC pathways, as well as the highest glycolytic and OXPHOS activity. At the DP early stage, glycolysis and mitochondrial respiration were invariably reduced in all murine and human systems, even in *Rag1* ^-/-^ M-ATOs. SP thymocytes exhibited metabolic gene expression and activity that was relatively increased compared to DP cells, but did not recover fully to the levels seen in early T cell development stages.

Transcriptional metabolic patterns and metabolic extracellular flux results in ATO-derived thymocytes were largely analogous to those of primary thymocytes. The fidelity of thymocyte metabolic dynamics *in vitro* is impressive, considering the differences in the microenvironment, including gas exchange, supportive tissue, and nutrient availability. Besides the lack of thymic epithelium, *in vitro* models are usually cultured in room air and media with supraphysiological cytokine concentrations, whereas the thymic medulla is thought to be hypoxic and nutrient availability is likely more localized (53). These findings suggest that, with the exception of Notch signaling, key metabolic changes during T cell development do not require the complex architecture of the primary thymus.

Despite a similar overarching pattern in development, some differences were noted between endogenous and *in vitro-*derived T cells. Our data suggests that thymocytes potentially utilize a higher fraction of their bioenergetic capacity at basal conditions *in vitro* compared to *in vivo*. This could be due to enriched media conditions. It is also possible that transcriptional differences seen in the DN1/Thy1 populations may reflect differences in HSPC origin. Although DN1 cells isolated from the thymus are transcriptionally closely related to their HSPC precursors in the marrow, exposure to the thymic microenvironment rapidly initiates transcriptional changes (12); HSPCs seeded in ATOs may be a more heterogeneous progenitor population, a minority of which are poised to launch T cell commitment (12, 27, 28). In addition, as described previously in the M-ATO, the global transcriptional profile in TCRβ^+^CD3^+^ “DP late” cells from ATOs partially overlaps with that of TCRβ^-^CD3^-^ “DP early” cells from primary thymus, suggesting earlier surface expression of TCRβ/CD3 on DP cells *in vitro*; this may explain the apparent mismatch in transcriptional downregulation of core metabolic genes at the DP late stage instead of the DP early stage in the ATO (27).

ETC gene expression and mitochondrial respiration were closely associated in the M-THY, M-ATO, and H-THY systems; meanwhile, the H-ATO showed uncoordinated ETC gene expression and yet a pattern of mitochondrial respiration consistent with all other systems. *MYC* expression appeared to also be dissociated with mitochondrial respiration specifically in DP early cells in the H-ATO system. On the other hand, mitochondrial mass was largely similar in H-THY and H-ATO cells. Although it is unclear why the human but not the mouse ATOs showed a lack of coordination between respiratory metabolism and ETC genes, additional regulatory mechanisms, including post-translational processes, may be controlling mitochondrial metabolism.

One key difference between the mouse and human systems may be the role of hypoxia, which has disparate effects on hematopoiesis and lymphopoiesis in mouse and human systems (54–57). Hypoxia has been shown to promote the development of human lymphoid progenitors from HSPCs, as well as T cell maturation from human HSPCs in the H-ATO (57, 58). Hence, differential *in vitro* oxygen requirements in human and mouse cells may be contributing to discrepant mitochondrial gene expression in the H-ATO.

Loss of *Rag1* had a deleterious impact on metabolism in the earlier DN stage of T cell development, consistent with previous reports of reduced glycolytic rates in *Rag2* ^-/-^ DN3 cells (59). On the other hand, the downregulation of basal respiratory and glycolytic activity at the DP early stage occurred regardless of the presence of TCR rearrangement. Therefore, metabolic downregulation in DP early cells is independent of TCR-mediated signaling.

“DP early” cells by definition do not express CD3 or TCR on the cell surface, so they are either precursors to the TCRβ^+^CD3^+^ DP late cells that have yet to undergo positive selection, or are DPs that will never generate a productive TCR complex and will eventually die by neglect (60). We only isolated live DP early cells for our metabolic analyses; nevertheless, we cannot exclude the possibility that some of the DP early cells studied were undergoing early apoptosis at the time of analysis. We did not see a significant difference in the proportion of early apoptotic cells between ISP and DP early populations, during which key metabolic transitions occur. Thus, it is unlikely that the dramatic changes in metabolism are solely due to global depression of transcription or metabolic activity secondary to apoptosis. An additional factor is the switching of glycolytic and TCA cycle isoenzyme gene expression during the transition from DN to DP, suggesting a transcriptionally driven metabolic fate decision.

Alterations in cytokine signaling pathways could be contributing to the highly specific metabolic downregulation in DP early cells. IL7RA is known to be down-regulated in murine and human DP cells (61, 62); however, DPs express high levels of other pro-survival cytokine receptors (63). Cytokine signal transduction has been shown to be suppressed in DP cells by suppressor of cytokine signaling (SOCS) and restored by TCR signaling (63). Thus, a combination of environmental signals as well as intrinsic mechanisms may be instructing DP early cells to undergo behavior reminiscent of growth factor deprivation in anticipation of low survival rates (64, 65). Moreover, the rise in metabolic activity in SP cells after the DP late stage could be due to functional TCR signaling that promotes the restoration of cytokine signaling sensitivity.

*NOTCH1* gene expression was highly associated with metabolic changes during development, supporting models that propose an interplay between Notch signaling and metabolic processes (13, 20, 59). While our analyses show a strong correlation between specific metabolic regulators and metabolic activity during thymocyte development in primary thymus or ATO systems, definitive experiments are required to study whether transcription factors such as NRF2 (*NFE2L2*) and STAT3/5 play an important role in controlling metabolism *in vitro*.

Though several studies have probed the mechanisms required for the DN-DP metabolic transitions in mice, further studies are needed to study the mechanisms and role of metabolic shifts in human thymocytes. This work contributes the thymocyte metabolism field by identifying critical species-conserved metabolic profiles in endogenous and *in vitro* murine and human thymic T cell differentiation.

## Data Availability Statement

RNA sequencing dataset is available from NIH's Sequence Read Archive (SRA) repository (PRJNA741323). A detailed description of data analysis and the software used can be found in Method details. The raw data and R code supporting the conclusions of this article will be available by the authors upon request.

## Ethics Statement

The animal study was reviewed and approved by UCLA Chancellor’s Animal Research Committee.

## Author Contributions

Conceptualization, VS, MS, GC, and UB. Methodology, VS and MS. Formal analysis, VS and MS. Investigation, VS, MS, AM-H, PC, AZ, SL, SB, and YZ. Data curation, VS, KK-U, and DC. Human thymus sample contribution, CP. Writing – original draft, VS and GC. Writing – review and editing, VS, MS, DC, SB, and GC. Supervision, GC. Funding acquisition, GC and UB. All authors contributed to the article and approved the submitted version.

## Funding

This work was supported by the National Institute of Health (NIH Bethesda, MD) grants RO1AG049753 (to GMC), 5P30AG028748/UL1TR000124 (NCATS/UCLA CTSI) (to DC), P30CA016042 (NCI) (TCGB and TPCL cores), NIH T32GM008042 (to VS), a BSCRC Innovation Award (to GMC, UB, MS), and the Connie Frank and Evan Thompson Program for Collaborative Restorative Transplantation Research (to GMC). This research was made possible by a grant to GMC from the California Institute of Regenerative Medicine (GC1R-06673-B [CRP]). The contents of this publication are solely the responsibility of the authors and do not necessarily represent the official views of CIRM or any other agency of the state of California. K.E.K-U acknowledges the support of the JCCC and Institute for Quantitative & Computational Biosciences at UCLA. VS acknowledges the support of the Eli and Edythe Broad Center of Regenerative Medicine and Stem Cell Research at UCLA Training Program.

## Conflict of Interest

AM-H and GC are listed on patents relating to this work. AM-H and GC are co-founders of PLUTO Immunotherapeutics Inc.

The remaining authors declare that the research was conducted in the absence of any commercial or financial relationships that could be construed as a potential conflict of interest.

## Publisher’s Note

All claims expressed in this article are solely those of the authors and do not necessarily represent those of their affiliated organizations, or those of the publisher, the editors and the reviewers. Any product that may be evaluated in this article, or claim that may be made by its manufacturer, is not guaranteed or endorsed by the publisher.

## Supplementary Material

Supplementary Table 1 (related to **Figure 1**): Population phenotypes for RNA-seq and Seahorse Assay Analyses.

Supplementary Table 2 (related to **Figure 2**): Differentially expressed genes in human thymus and ATO RNA-seq dataset listed in order of appearance as shown in **Figure 2B**.

Supplementary Table 3 (related to **Figure 5** **,** **6**, **Supplementary Figure 4**, **5**): Results from correlation analyses between extracellular flux and transcriptional data in mouse and human thymocytes.

Supplementary Table 4 Key Resource Table.

## Abbreviations

ATO, artificial thymic organoid; M-THY, mouse thymus; H-THY, human thymus; M-ATO, mouse ATO; H-ATO, human cord blood ATO; HSPC, hematopoietic stem and progenitor cell; iPSC, induced human pluripotent stem cell; DN, double negative; ISP, immature single positive; DP, double positive; SP, single positive; TCR, T cell receptor; PCA, principal component analysis; GO, gene ontology; ETC, electron transport chain; OXPHOS, oxidative phosphorylation; TCA, tricarboxylic acid cycle; FAO, fatty acid beta-oxidation; PPP, pentose phosphate pathway; OCR, oxygen consumption rate; ECAR, extracellular acidification rate; SRC, spare respiratory capacity; RAG, recombination activating gene.
