# Supplementary figures and images for: The Metabolic Landscape of Thymic T Cell Development In Vivo and In Vitro
Source: Front Immunol. 2021 Jul 28;12:716661. doi: 10.3389/fimmu.2021.716661 (PMC8355594; doi:10.3389/fimmu.2021.716661)

# Supplementary Figure 1

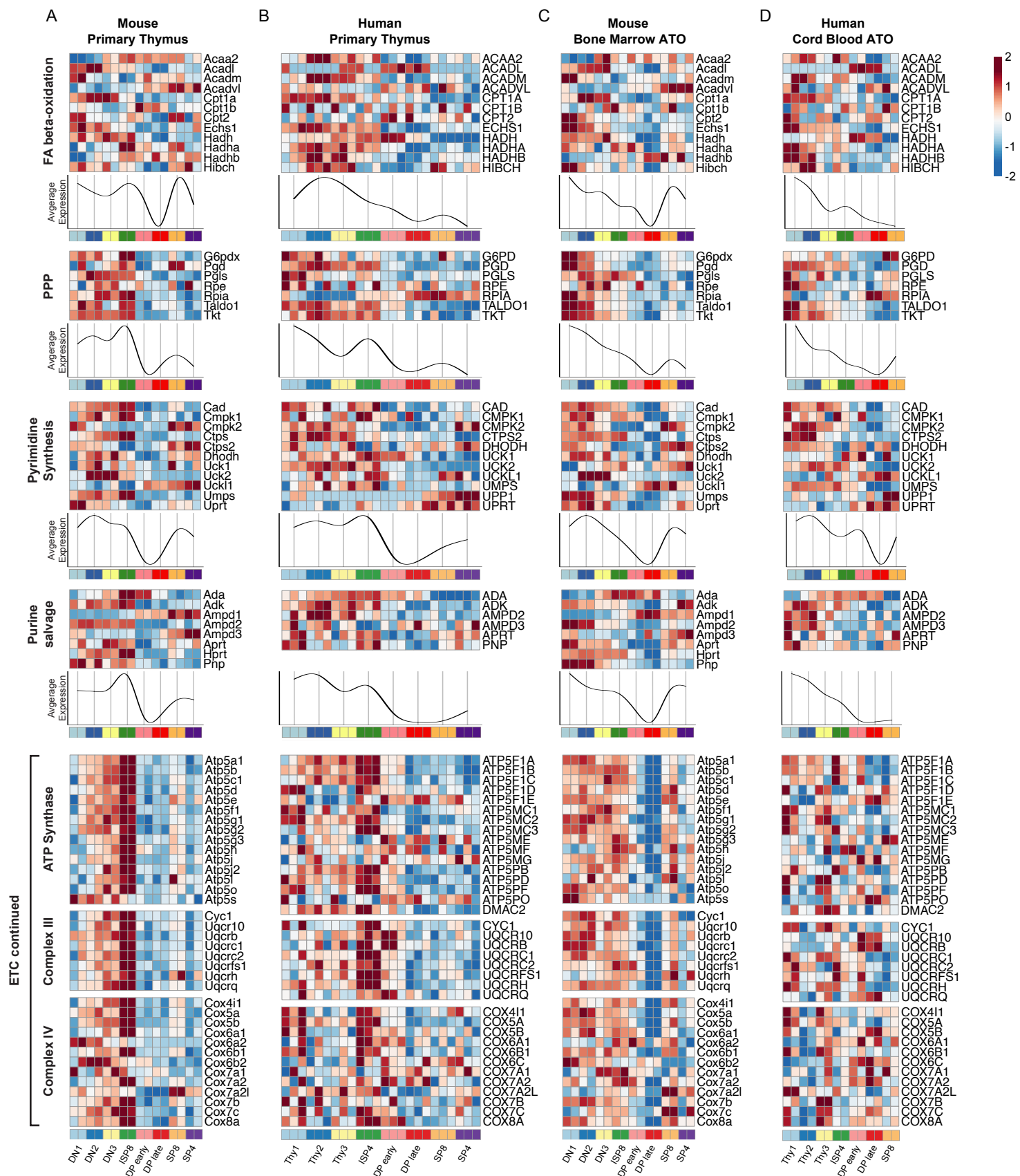

Supplement: Supplementary file 5 [file Image_1.pdf]
