# Supplementary material for: The Metabolic Landscape of Thymic T Cell Development In Vivo and In Vitro
Source: Front Immunol. 2021 Jul 28;12:716661. doi: 10.3389/fimmu.2021.716661 (PMC8355594; doi:10.3389/fimmu.2021.716661)
Supplement: Supplementary file 6 [file Image_2.pdf]

Supplementary Figure 2

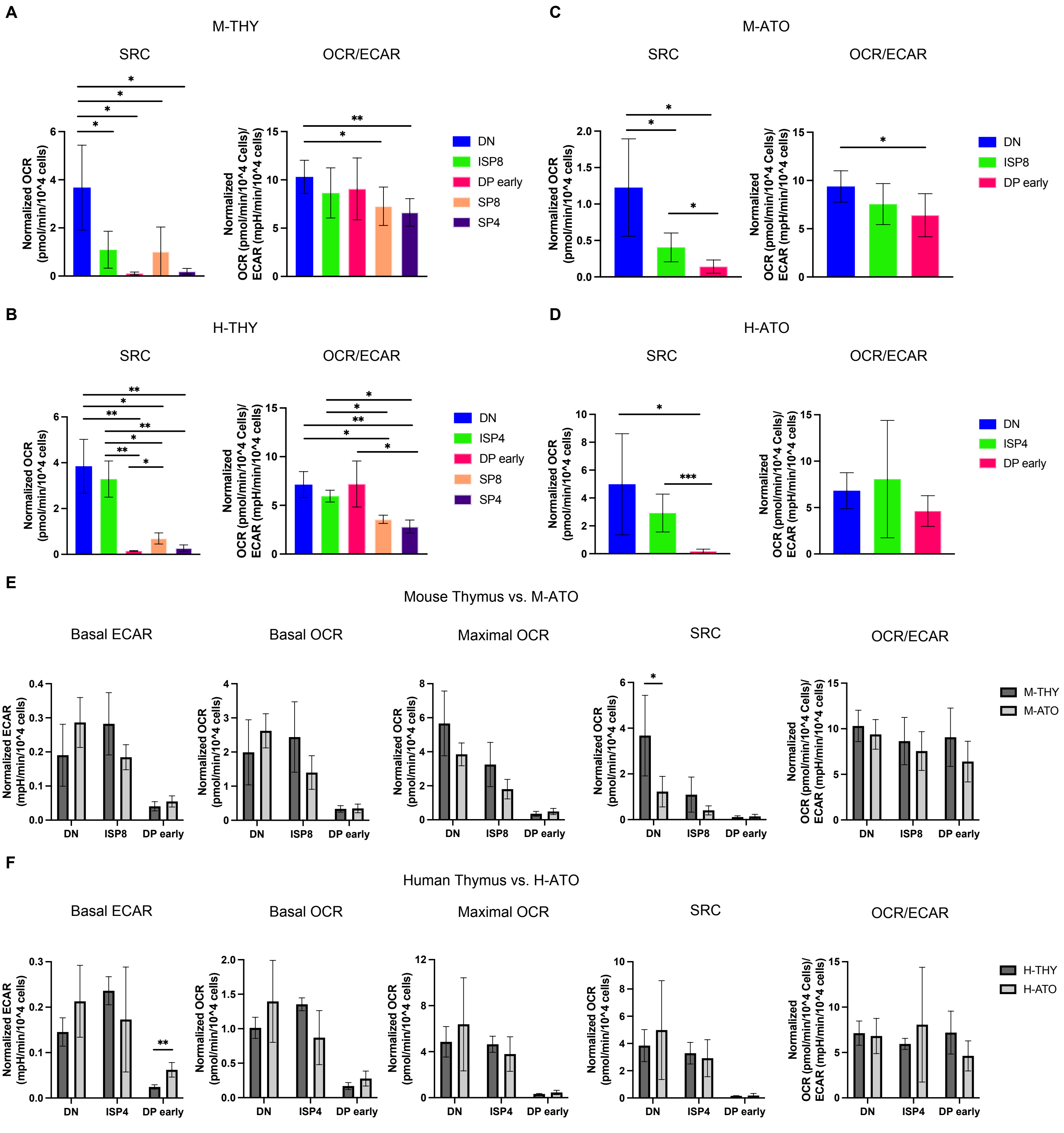

Supplementary Figure 2 (related to Figure 4):

(A-D) SRC and OCR/ECAR ratio in thymocyte populations from **(A)** M-THY, **(B)** H-THY, **(C)** M-ATO, and **(D)** H-ATO. Measurements are normalized to cell number per well. (n=6 independent experiments in M-THY; n=6 MATO; n=3 H-THY [except for ISP4: n=2]; n=6 H-ATO [except for ISP4: n=5])

(**E**) Extracellular flux measurements are compared between M-THY and M-ATO. (n=6 independent experiments in M-THY; n=6 MATO)

(**F**) Extracellular flux measurements are compared between H-THY and H-ATO. (n=3 H-THY [except for ISP4: n=2]; n=6 H-ATO [except for ISP4: n=5])

(error bars: sd, significance: multiple t-test p values * p <0.05; ** p<0.01, *** P<0.001)
