# Supplementary material for: The Metabolic Landscape of Thymic T Cell Development In Vivo and In Vitro
Source: Front Immunol. 2021 Jul 28;12:716661. doi: 10.3389/fimmu.2021.716661 (PMC8355594; doi:10.3389/fimmu.2021.716661)
Supplement: Supplementary file 7 [file Image_3.pdf]

Supplementary Figure 3

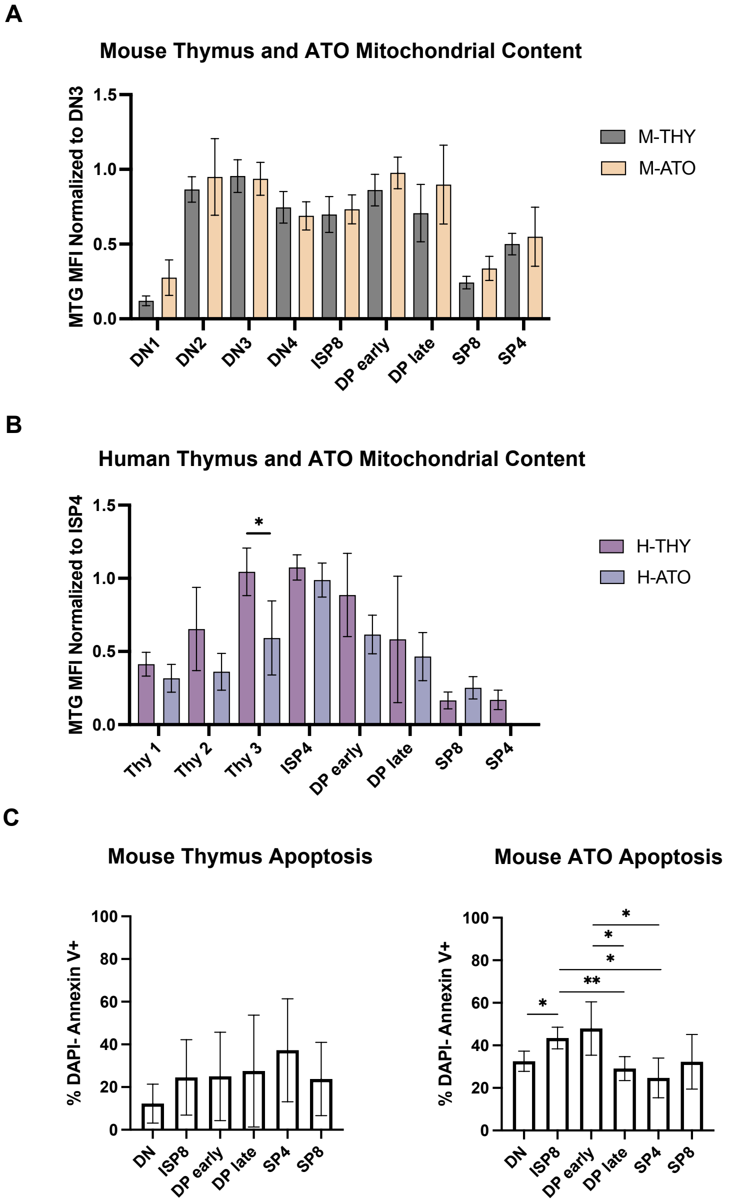

Supplementary Figure 3 (related to Figure 4):

(A-B) Mitochondrial content measurements using Mitotracker Green (MTG) in **(A)** M-THY and M-ATO, or **(B)** H-THY and H-ATO. MTG mean fluorescence intensity (MFI) was normalized to DN3 thymocytes in each experiment in M-THY and M-ATO, while MTG MFI was normalized to ISP4 thymocytes in each H-THY and H-ATO experiment. (n=6 independent experiments in M-THY, n=3 M-ATO, n=4 H-THY, n=6 CB-ATO).

(C) Proportion of early apoptotic cells (DAPI- Annexin V+) in M-THY and M-ATO thymocyte populations. (n=3 independent experiments in M-THY; n=4 M-ATO).

(error bars: sd, significance: multiple t-test p values * $p < 0.05$; ** $p < 0.01$)
