# Supplementary material for: The Metabolic Landscape of Thymic T Cell Development In Vivo and In Vitro
Source: Front Immunol. 2021 Jul 28;12:716661. doi: 10.3389/fimmu.2021.716661 (PMC8355594; doi:10.3389/fimmu.2021.716661)
Supplement: Supplementary file 8 [file Image_4.pdf]

Supplementary Figure 4

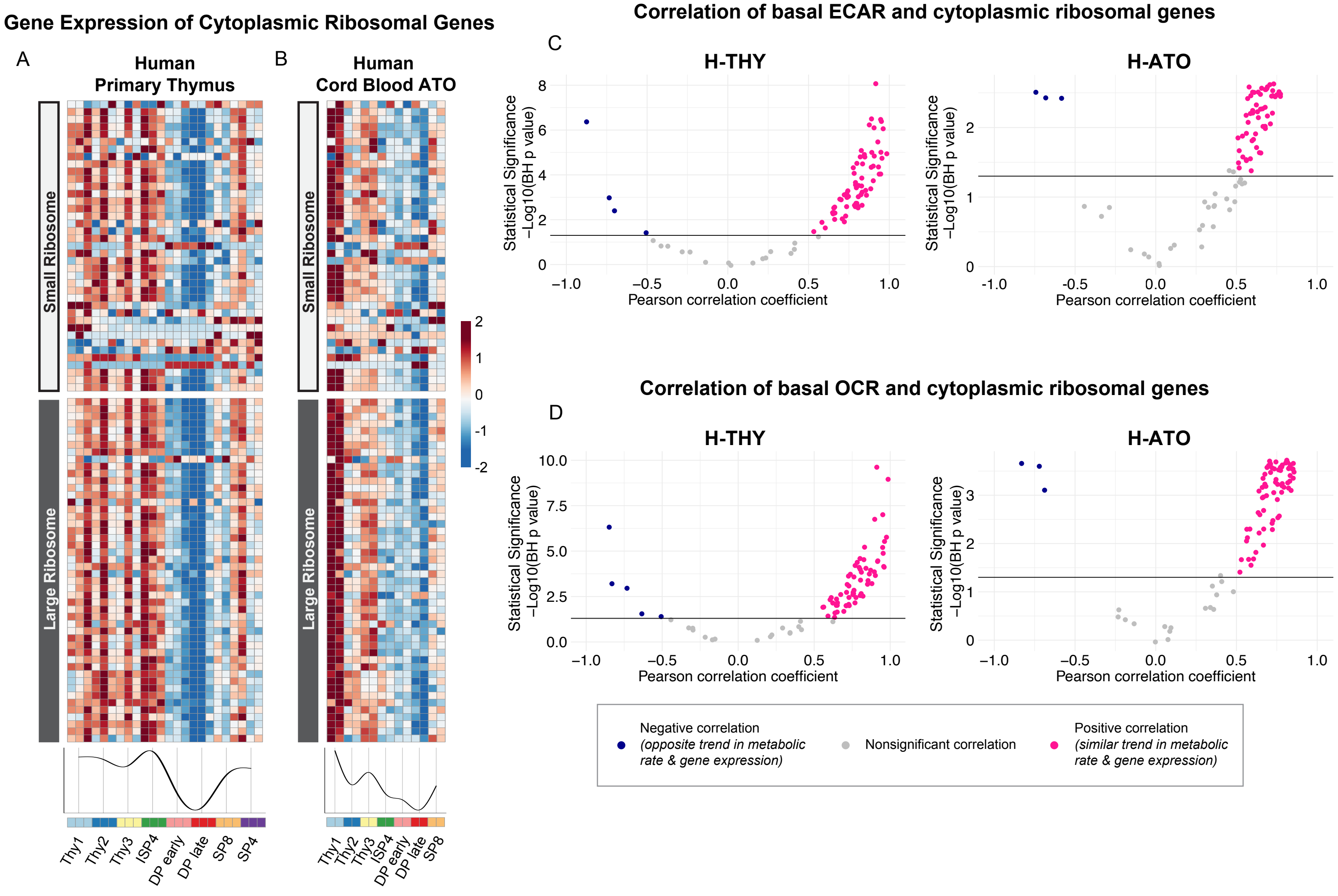

Supplementary Figure 4 (related to Figure 6):

(A-B) Summary heatmaps and line plots of cytoplasmic ribosomal genes (small and large ribosome) in **(A)** H-THY and **(B)** H-ATO. The x axis indicates cells isolated for analysis. Each individual heatmap represents *z*-scores of normalized variance-stabilized gene expression data. Line plots represent the average *z*-score for all genes in the heatmap. (n=3 in H-THY; n=2 in H-ATO)

(**C-D**) Scatter plots show correlation between (**C**) small/large cytoplasmic ribosomal genes and basal glycolysis (ECAR) or (**D**) small/large cytoplasmic ribosomal genes and basal respiration (OCR) in the H-THY (left) or H-ATO (right). The x axis represents the Pearson correlation coefficient R values calculated for individual genes within the specified metabolic pathway for all thymocyte populations for which respective extracellular flux data was collected. The y axis represents the statistical significance of each R value using -log10 of Benjamini-Hochberg adjusted p-values. Solid black line indicates an adjusted p-value of 0.05; all R values above the line are statistically significant. Genes with R values >0.5 (pink) are positively correlated with metabolic flux data, while genes with R values <-0.5 (blue) are negatively correlated with metabolic flux data. Nonsignificant or weak correlation R values >-.05 or <0.5 are indicated in grey. (H-THY: n=3 flux analysis values for 5 populations [except for ISP4: n=2], n=3 RNA-seq replicates; H-ATO: n=6 flux analysis values for 3 populations [except for ISP4: n=5], n=2 RNA-seq replicates)
