# Supplementary material for: The Metabolic Landscape of Thymic T Cell Development In Vivo and In Vitro
Source: Front Immunol. 2021 Jul 28;12:716661. doi: 10.3389/fimmu.2021.716661 (PMC8355594; doi:10.3389/fimmu.2021.716661)
Supplement: Supplementary file 9 [file Image_5.pdf]

Supplementary Figure 5

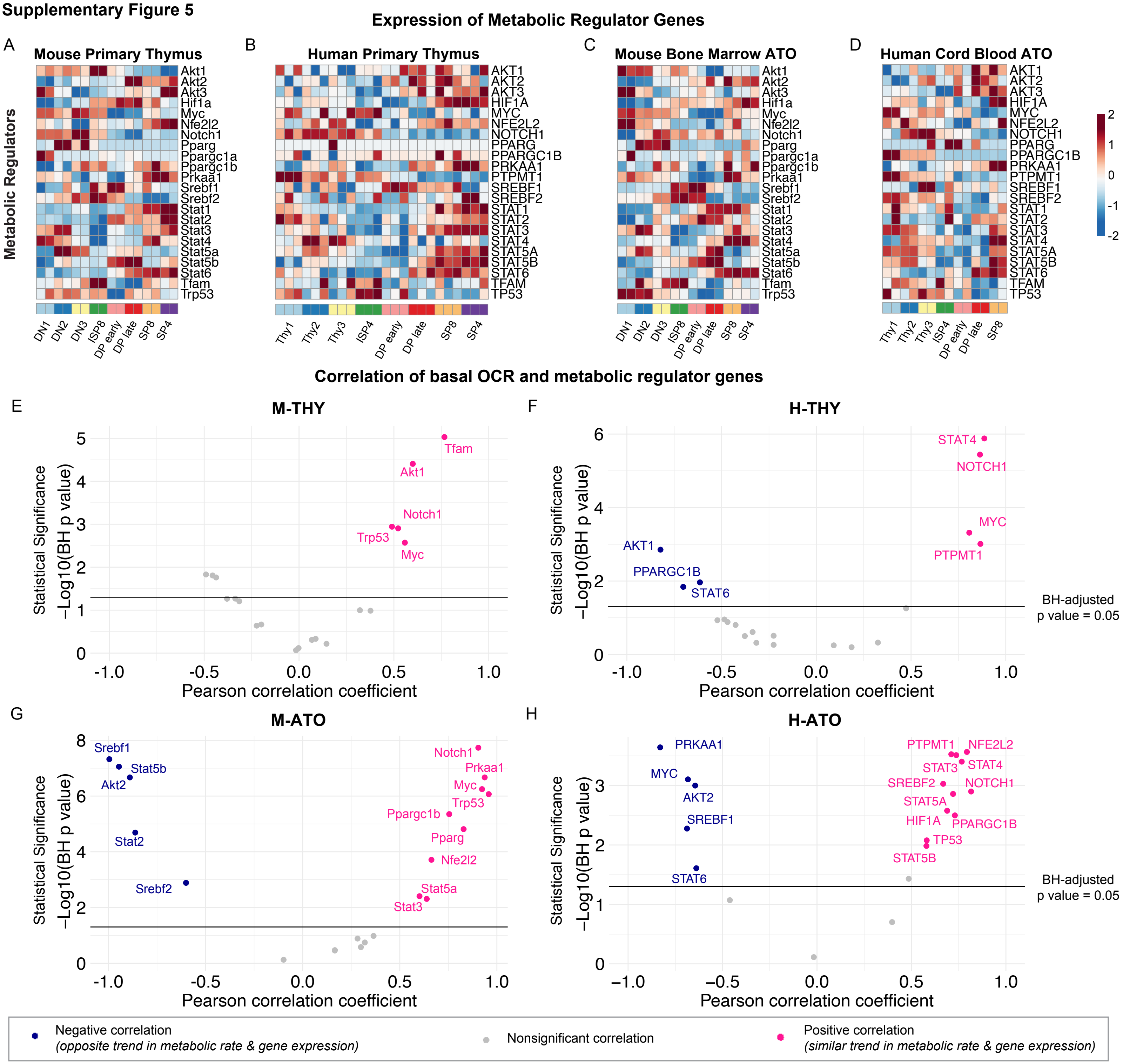

Supplementary Figure 5 (related to Figure 6):
(A-D) Heatmaps and summary line plots of genes encoding key signaling molecules and transcription factors that regulate metabolic programs in **(A)** M-THY, **(B)** H-THY, **(C)** M-ATO, and **(D)** H-ATO. The x axis indicates cell populations isolated for analysis. Each individual heatmap represents *z*-scores of normalized variance-stabilized gene expression data. (n=2 independent replicates per population in M-THY; n=3 in H-THY; n=2 in M-ATO; n=2 in H-ATO).
(E-H) Scatter plots show correlation between gene expression of metabolic regulators and basal respiration (OCR) in (**E**) M-THY, (**F**) H-THY, (**G**) M-ATO, and (**H**) H-ATO. The x axis represents the Pearson correlation coefficient R values calculated for individual genes within the specified metabolic pathway for all thymocyte populations for which respective extracellular flux data was collected. The y axis represents the statistical significance of each R value using -log10 of Benjamini-Hochberg adjusted p-values. Solid black line indicates an adjusted p-value of 0.05; all R values above the line are statistically significant. Genes with R values >0.5 (pink) are positively correlated with metabolic flux data, while genes with R values <-0.5 (blue) are negatively correlated with metabolic flux data. Nonsignificant or weak correlation R values >-.05 or <0.5 are indicated in grey. (M-THY: n=6 flux analysis values for 5 populations, n=2 RNA-seq replicates; H-THY: n=3 flux analysis values for 5 populations [except for ISP4: n=2], n=3 RNA-seq replicates; M-ATO: n=6 flux analysis values for 3 populations, n=2 RNA-seq replicates; H-ATO: n=6 flux analysis values for 3 populations [except for ISP4: n=5], n=2 RNA-seq replicates)
